# In vitro characterization of hydroxyapatite and cobalt ferrite nanoparticles compounds and their biocompatibility in vivo

**DOI:** 10.1007/s10856-022-06640-z

**Published:** 2022-02-07

**Authors:** Cristiane C. Vital Cintra, Dayana A. C. Ferreira-Ermita, Fabrícia H. Loures, Pascally M. A. G. Araújo, Iara M. Ribeiro, Fabiana R. Araújo, Fabrício L. Valente, Emily C. Carlo Reis, Ana Cristina F. M. Costa, Sheila M. C. M. Bicalho, Andréa P. B. Borges

**Affiliations:** 1 Veterinary Department, Universidade Federal de Viçosa, Viçosa, Brasil; 2 Laboratory of Synthesis of Ceramic Materials, Universidade Federal de Campina Grande, Campina Grande, Brasil; 3 Veterinary Department, Universidade Federal de Minas Gerais, Belo Horizonte, Brasil; 4 JHS Chemical Laboratory Ltda, Sabará, Brasil

**Keywords:** Biomaterial, Cobalt ferrite nanoparticles, Hydroxyapatite, Biocompatibility

## Abstract

Bioactive materials in combination with antibiotics have been widely developed for the treatment of bone infection. Thus, this work aims to characterize six biomaterials formulated with different concentrations of hydroxyapatite and cobalt ferrite nanoparticles, in addition to the antibiotic ciprofloxacin, using X-ray diffraction (XRD), scanning electron microscopy (SEM), and the antibiotic diffusion test on agar. Furthermore, in vivo biocompatibility and the reabsorption process of these materials were analyzed. XRD showed that both hydroxyapatite and cobalt ferrite present high crystallinity. The photomicrographs obtained by SEM revealed that composites have a complex surface, evidenced by the irregular arrangement of the hydroxyapatite and cobalt ferrite granules, besides demonstrating the interaction between their components. The antibiotic-diffusion test showed that all biomaterials produced an inhibition halo in *Staphylococcus aureus* cultures. For the biocompatibility study, composites were surgically implanted in the dorsal region of rabbits. At 15, 30, 70, and 100 days, biopsies of the implanted regions were performed. The biomaterials were easily identified during histological analysis and no significant inflammatory process, nor histological signs of toxicity or rejection by the adjacent tissue were observed. We can conclude that the biomaterials analyzed are biocompatible, degradable, and effective in inhibiting the in vitro growth of *Staphylococcus aureus*.

**Figure:**
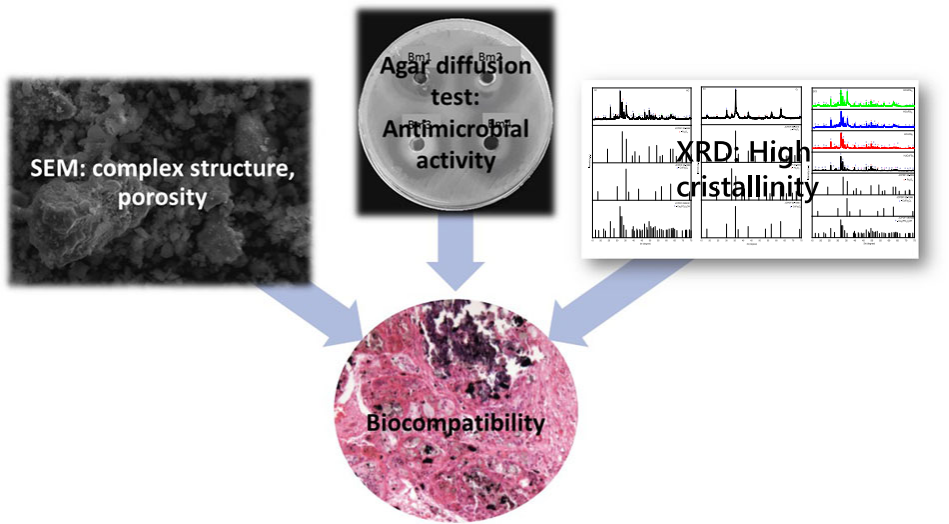
Graphical abstract

Graphical abstract

## Introduction

Biomaterials are substances of natural or synthetic origin, used in biomedical applications and that interact with biological systems, treating, increasing, or replacing tissues, organs, or restoring functions compromised by degenerative processes or trauma [1]. Due to their importance in the medical field, the development of new types of biomaterials has been highlighted, leveraging studies related to tissue engineering or bioengineering that uses knowledge in the areas of materials science, biology, chemistry, medicine, and engineering to develop new products that restore, maintain or improve tissue function [2, 3].

Especially in orthopedics and dentistry, the use of biomaterials to repair damaged parts of the bone has been revolutionary [4–6]. The group of biomaterials that most resemble bone composition is the group of calcium phosphate ceramics [7, 8], among which the most widespread is hydroxyapatite (HAp) [4]. It has been used because it is a bioactive, nontoxic substance that causes little tissue reaction [9–11] and is biocompatible with bone, once both have the same type of crystalline structure [3, 10, 11]. Besides that, it has a porous structure, which is an advantage for bone ingrowth [12–14].

Knowing a biomaterial’s crystallinity is important considering that it influences in vivo performance as it affects the resorption and its mechanical properties, since amorphous regions are reabsorbed faster than crystalline regions [15]. Porosity, on the other hand, is related to the angiogenesis, which is an important step for tissue regeneration after an injury. The porous structure of HAp works as a passive support for the angiogenesis process to occur. At this stage, a sequence of events is initiated to repair injured tissues, in which new blood vessels are formed from preexisting vessels [16–18]. HAp provides an additional substrate for this process to occur, leading to the proliferation of bone tissue [19–21], and allowing the proliferation, migration, and phenotypic expression of bone cells, i.e., it is osteoconductive [15, 22]. In addition to biomaterials presenting a microstructure of interconnected pores, they must also have surface complexity (surface roughness and topography) suitable for tissue growth. These factors influence cell adhesion and activity, and angiogenesis, that is, they are directly related to the biocompatibility of the material [15, 23].

Iron oxide-based nanoparticles have been used for biomedical applications for more than 10 years [24], as they feature magnetic moment dependent on nanoparticle size, high colloidal stability, simple surface functionality, chemical stability, and reactive surfaces to attach biological molecules [25, 26]. Besides that, magnetic iron oxide nanoparticles show excellent biocompatible, biodegradable, and nontoxic properties [24]. Currently, various iron oxide systems in biological applications have been reported. Among the most common, magnetite (FeFe_2_O_4_) and hematite (Fe_2_O_3_) stand out for presenting nontoxic characteristics and magnetizing force suitable for biological applications [26]. In the magnetite category, which presents a spinel-like crystalline structure, cobalt ferrite (CoFe2O4) has been widely researched due to its low-toxicity characteristics, excellent water solubility, physiological pH stability, and magnetic behavior suitable for use in biosensors [27]. CoFe_2_O_4_ has been used in several biomedical applications as a catalyst, in hyperthermia treatment, magnetic resonance imaging, and in biosensors, among others. According to Amiri and Shokrollahi [28], CoFe_2_O_4_ can be functionalized with drugs and applied in drug-delivery systems (DDS), especially in the area of chemotherapy, and treatment of various infections.

As a biocompatible material, HAp combined with cobalt ferrite nanoparticles is a promising device for drug delivery and regeneration of bone tissue [29, 30]. From this context, the objective of this study was to characterize the biomaterials formulated with HAp and CoFe_2_O_4_ and evaluate their biocompatibility and resorption processes aiming at the subsequent use of these materials in bone-regeneration processes in cases of infection.

## Materials and methods

### Ethical aspects

All procedures adopted in this work are in accordance with the Code of Professional Ethics of Veterinarians and the Ethical Principles in Animal Experimentation adopted by the Brazilian College of Animal Experimentation (COBEA) and with the current legislation. The methodology was approved by the Ethics Committee on Animal Use of Federal University of Viçosa (CEUA/UFV), under the protocol 67/2016.

### Biomaterial

#### Materials used

- Magnetic nanoparticles (MNPs) of cobalt ferrite (CoFe_2_O_4_) obtained by the combustion-reaction technique in batches of 10 g of product/reaction.
- Hydroxyapatite (HAp) obtained through the precipitation process and calcined at 900 °C according to the methodology proposed by Medvecky et al. [31].
- Commercial hydroxyapatite produced by JHS Ltda laboratory (HAP 91^®^).

#### Preparation of biomaterials

The methodology proposed by Araújo [27] was used to obtain the biomaterials. The procedure is summarized as follows: (i) 2.5 g of CoFe_2_O_4_ MNPs and 100 mL of ethanol, under ultrasonic agitation for 30 min, were coated with TEOS (tetraethylorthosilicate) and APTS (3-aminopropyltrimethoxysilane), resulting in a core–shell-type structure with reactive -OH and NH_2_ groups; (ii) after obtaining the CoFe_2_O_4_@SiO_2_ hybrid, it was physically mixed with hydroxyapatite (HAp), resulting in a HAp:CoFe_2_O_4_@SiO_2_ composite. Then, ciprofloxacin was incorporated at a mass concentration of 10% in hybrid materials. The different compositions and mass proportions of the biomaterials produced are described in Table 1. All the biomaterials were prepared in the Ceramic Materials Synthesis Laboratory (LABSMAC) of the Federal University of Campina Grande (UFCG).

**Table 1:** List of biomaterials, expressed in symbology and mass ratio

Codes	Mass ratio % and composition	m_HAp_ (g)	m_CoFe2O4_ (g)	mγ (g)	m_T_ (g)
HCoFSγ_1_	HAp: CoFe_2_O_4_@SiO_2_ (70:30)+γ	17,5	7,5	2,5	27,5
HCoFSγ_2_	HAp: CoFe_2_O_4_@SiO_2_(50:50)+γ	12,5	12,5	2,5	27,5
HCoFSγ_3_	HAp: CoFe_2_O_4_@SiO_2_ (30:70) +γ	7,5	17,5	2,5	27,5
JCoFSγ	HAp JHS: CoFe_2_O_4_@SiO_2_ (70:30)+γ	12,6	5,4	1,8	19,8
Cγ	CoFe_2_O_4_@SiO_2_ + γ	–	25,0	2,5	27,5
HC	HAp: CoFe_2_O_4_@SiO_2_ (70:30)	11,2	4,8	–	16,0

HAp: hydroxyapatite; CoFe2O4@SiO2: coated cobalt ferrite; γ: ciprofloxacin; HApJHS: hydroxyapatite (JHS laboratory); mHAp: mass in grams of hydroxyapatite; mCoFe2O4: mass in grams of cobalt ferrite; m(: mass in grams of ciprofloxacin; MT: total mass in grams

### Characterization

Scanning electron microscopy (SEM) and X-ray diffraction (XRD) techniques were used for the biomaterials’ characterization. Surface topography, morphology, as well as the presence of clusters and pores were analyzed by SEM using a LEO 1430VP microscope at a voltage of 15 kV. The compound surfaces were coated with gold and images were taken at 200x, 500x, 1000x, 2000x, and 30000x magnifications, from which qualitative data of the surface topography of the materials were obtained. The present phases, the crystallinity and crystallite size in the samples, were determined from X-ray diffraction (XRD) data obtained with a D2 Phaser, Bruker diffractometer; radiation CuKα. The average crystallite size was determined from the Scherrer equation and the crystallinity was determined from the ratio between the integrated peak area for the crystalline phase and the area for the amorphous fraction.

### Agar antibiotic-diffusion test

The biomaterials were tested for their antibacterial activity against *Staphylococcus aureus* using the agar-diffusion assay. The test was carried out according to the principle of drug diffusion, using a disk containing ciprofloxacin (positive control) and the compressed biomaterials in the form of 5 mg tablets.

A bacterial inoculum was prepared from the stock culture of *Staphylococcus aureus* (*S. aureus*) strains, maintained at −80 °C, from the Laboratory of Bacterial Diseases of the Department of Veterinary Medicine of the Federal University of Viçosa (UFV) and with laboratory-proven virulence. A small aliquot of the frozen culture was transferred to the Brain and Heart Infusion (BHI) broth with the aid of an autoclaved wooden stick under sterile conditions. The tube with the inoculated broth was placed in an oven at 37 °C for 18–24 hours. Turbidity was adjusted to D.O.550 0,08–0,1 (equivalent to McFarland 0,5 scale), resulting in a suspension containing approximately 1–2 × 108 CFU/mL.

After obtaining the inoculum, two Petri dishes containing Mueller–Hinton agar were sown with the bacterial culture. Then, holes were produced in the agar with the aid of a sterile pipette tip. Subsequently, the sterile biomaterial tablets and the disk containing ciprofloxacin were placed in these holes. The test was performed in duplicate. After the application of the biomaterials and the disk, the plates were incubated under aerobiosis for 24 hours at 37 °C. The reading was performed by measuring the diameters of the inhibition halos formed around the biomaterial “tablets” and the disk containing ciprofloxacin and obtaining the mean between the halos formed in three tests.

### Biocompatibility analysis of biomaterials in vivo

Six New Zealand rabbits from the rabbit breeding sector of Animal Science Department at UFV were randomly chosen for in vivo biocompatibility analysis. In order to implant the biomaterials, the animals were sedated using an intramuscular association of the drugs Ketamine and Midazolam at doses of 12.5 mg/kg and 1 mg/kg, respectively. Then, anesthetic induction and maintenance were performed using the inhalation drug isoflurane in 100% oxygen administered through a mask.

One animal was used for each biomaterial to be implanted, and these were surgically implanted in quadruplicate, in the dorsal region, between the fascia and the muscle tissue of each rabbit. Immediately before being implanted, each material, in the approximate amount of 2.0 mg, was moistened with 0.5 mL of sterile saline solution in order to form a moldable paste. The paste was then molded into a 5 mm-diameter tablet, which was then implanted as previously described. After implantation, the tissues were sutured with 2–0 nylon.

The animals were submitted to daily observations of the surgical wound for one week after the surgical procedure, assessing the degree of inflammatory reaction based on the presence of edema and pain, by visual inspection and digital pressure of the operated area. The presence of hemorrhage, purulent secretion, and dehiscence of the suture was also visually evaluated. At 15, 30, 70, and 100 days, a sample of each animal was obtained for routine histological processing (10% formaldehyde fixation, dehydration, paraffin inclusion, microtomy, and hematoxylin–eosin staining). Fragments of approximately 2.0 × 2.0 cm were collected, containing skin, subcutaneous tissue, fascia, muscle tissue, and biomaterial. On the 100th day (last harvest), the animals were euthanized by anesthetic overdose.

The presence of the biomaterial, its morphology, tissues and cells in contact with the biomaterial, and the presence or absence of a fibrous capsule and its thickness, when present, were evaluated and described. Hyperemia, inflammatory infiltration, presence of hemorrhagic regions, tissue infiltration within the biomaterial, and evidence of phagocytosis were also evaluated and described. Regarding fibroplasia, hyperemia, and inflammatory infiltration, the slides were evaluated in relation to the intensity of the alterations, being classified as slight, moderate, or intense.

## Results

### Characterization

**Fig. 1:**
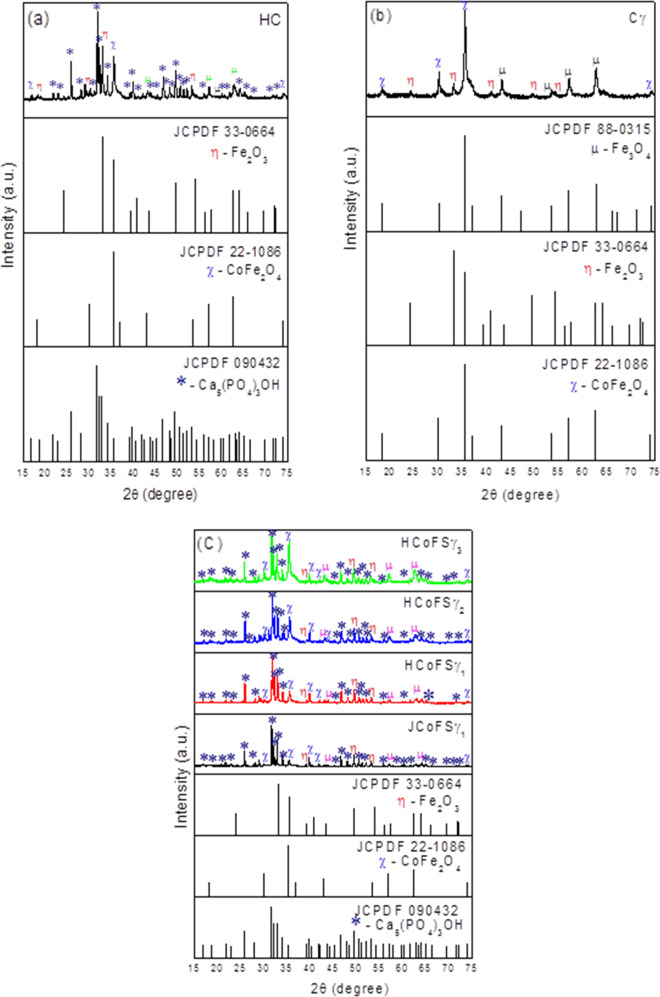
X-ray diffraction (XRD) of the hybrid composites: (**a**) CoFe_2_O_4_@SiO_2_:HAp (HC); (**b**) CoFe_2_O_4_@SiO_2_:γ (Cγ); (**c**) CoFe_2_O_4_@SiO_2_:Hap (70:30) (JCoFSγ_1_); CoFe_2_O_4_@SiO_2_:HAp (70:30) (HCoFSγ_1_); CoFe_2_O_4_@SiO_2_:HAp (50:50) (HCoFSγ_2_) e CoFe_2_O_4_@SiO_2_:HAp (30:70) (HCoFSγ_3_)

Figure 1 illustrates the X-ray diffraction graphs of hybrid composite HC (Fig. 1a), hybrid composite Cγ (Fig. 1b), and of hybrid composites JCoFSγ_1_; HCoFSγ_1_; HCoFSγ_2_, and HCoFSγ_3_, (Fig. 1c). In Fig. 1a, for the hybrid composite HC, the presence of four phases can be observed, with the main phase of HAp Ca_5_(PO_4_)_3_OH, according to the standard form JCPDF 09-0432 and the second phase of traces of CoFe_2_O_4_, corresponding to the crystallographic card JCPDF 22-1086, Fe_2_O_3_ referring to the crystallographic card 33-0664, and peaks referring to Fe_3_O_4_ in 62.63; 57.21, and 43.20° corresponding to the crystallographic card 88-0315. Figure 1b shows the formation of the phase of CoFe_2_O_4_ and Fe_2_O_3_ ferrite (hematite) and traces of Fe_3_O_4_ magnetite identified by means of the crystallographic cards JCPDF 22-1086, JCPDF 33-0664, and JCPDF 88-0315, respectively.

It can be observed in the diffractograms of Fig. 1c, for the hybrid composite synthesized with the commercial hydroxyapatite from JHS Biomaterials (JCoFSγ_1_) in the concentration of 70:30 HAp:CoFe_2_O_4_@SiO_2_ and in the systems HCoFSγ_1_; HCoFSγ_2_, and HCoFSγ_3_ in the concentrations of 70:30; 50:50, and 30:70, in general, the presence of four phases. The main phase of HAp Ca_5_(PO_4_)_3_OH, according to the standard plug JCPDF 09-0432 and the second phase of traces of CoFe_2_O_4_, Fe_2_O_3_ corresponding to the crystallographic plugs JCPDF 22-1086, 33-0664,and peaks of Fe_3_O_4_ around 62.63; 57.21; and 43.20° referring to Fe_3_O_4_ corresponding to the crystallographic plug 88-0315.

In Table 2, the crystallinity and crystallite size of hybrid composites (HC, Cγ, JCoFSγ_1_, HCoFSγ_1_, HCoFSγ_2_, and HCoFSγ_3_) are described.

**Table 2:** Crystallinity and crystallite size of hybrid composites

Samples	Crystallinity (%)	Crystal size (nm)
Cγ	66.00	31.00
HC	76.40	54.10
JCoFSγ_1_	90.40	69.70
HCoFSγ_1_	74.99	51.14
HCoFSγ_2_	89.70	37.32
HCoFSγ_3_	86.90	37.49

**Fig. 2:**
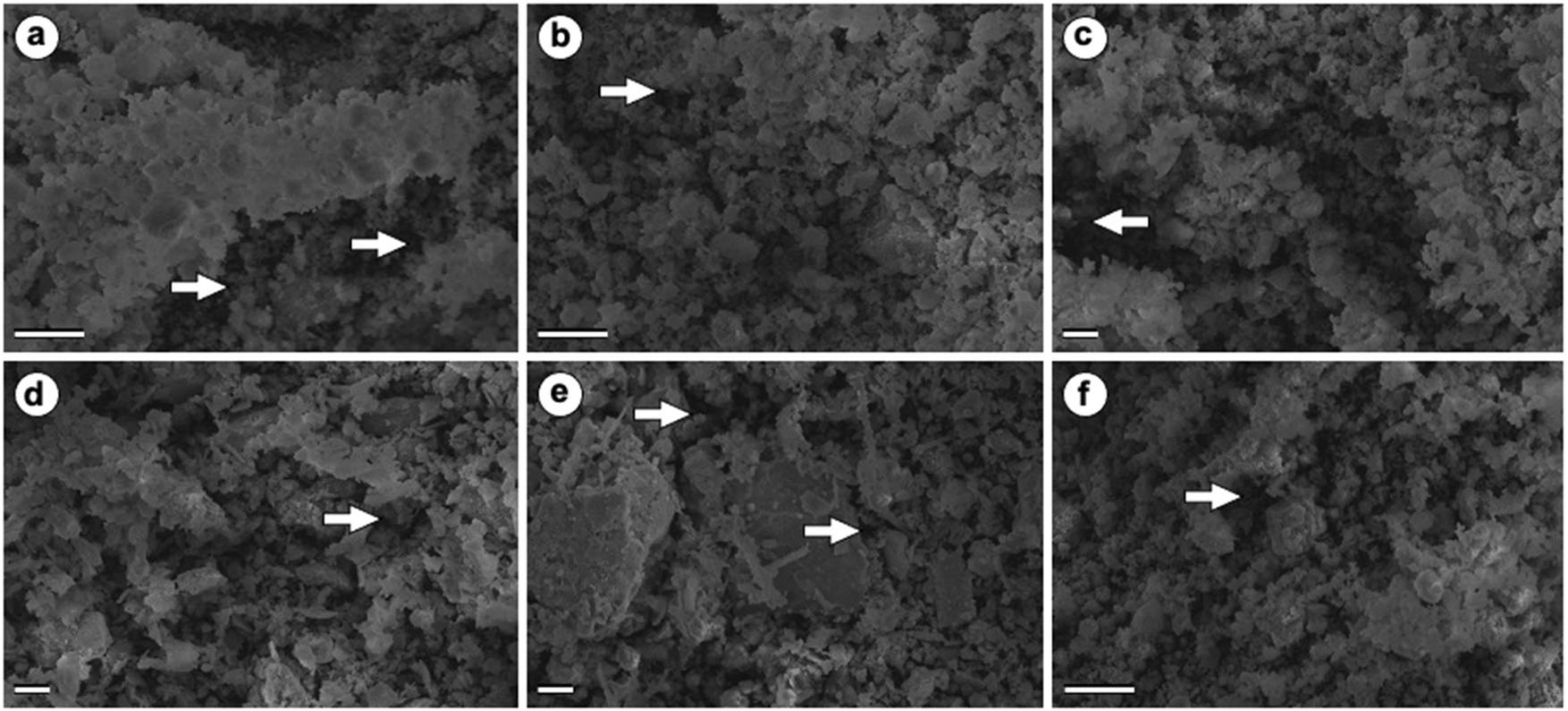
Photomicrographs of scanning electron microscopy of biomaterials. Bars: 20 µm. **a** HCoFSγ_1_ (70:30) (**b**) HCoFSγ_2_ (50:50); (**c**) HCoFSγ_3_ (30:70); (**d**) JCoFSγ (70:30); (**e**) Cγ; and (**f**) HC (70:30). The arrows highlight the pores

Figure 2 illustrates the photomicrographs obtained by SEM of biomaterials. Crystals in various formats of hydroxyapatite granules and cobalt ferrite can be observed, characterizing a complex surface topography, and showing the interaction between material components. In all the SEM images, it is possible to observe a structure composed of heterogeneous and highly agglomerated grains and clusters of irregular size. The existence of pores in the biomaterials was also observed.

As can be seen from the photos, it is not possible to differentiate between HAp and cobalt ferrite particles. Although they are not exactly the same, they have very similar structure (size and shape of the granules), making their differentiation difficult due to their SEM appearance. Nanosized CoFe_2_O_4_ particles could not be detected.

### Agar-diffusion test

**Fig. 3:**
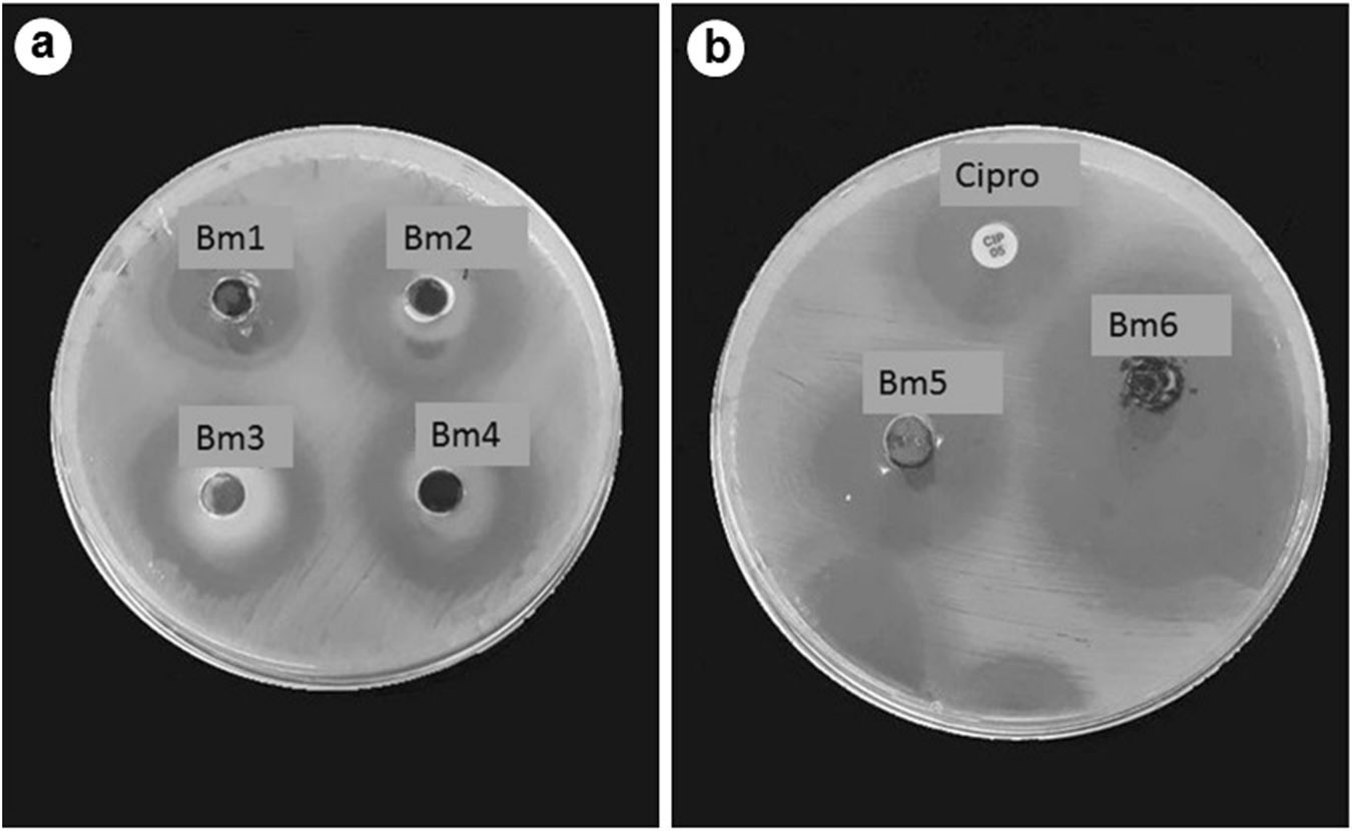
Petri dishes showing the halos formed in the agar-diffusion test. Bm1: HC; Bm2: HCoFSγ_2_; Bm3: HCoFSγ_1_; Bm4: HCoFSγ_3_; Bm5: JCoFSγ_1_; Bm6: Cγ; Cipro: ciprofloxacin disk

The halos resulting from the diffusion test are described in Table 3 and can be seen in Fig. 3. The values presented are the mean of the halos observed in each test, by the biomaterial tested. Despite not having ciprofloxacin in its composition, there was formation of a halo around the biomaterial HC, and the average value of these halos was 20.5 mm.

**Table 3:** Biomaterials and the mean diameters of the halos formed around each of them

Material	Mean
Cipro	22.25
HCoγ_1_	30.25
HCoγ_2_	30
HCoγ_3_	26.5
JCoγ	23.75
HC	20.75
Cγ	32.5

Cipro: ciprofloxacin disc

### In vivo biocompatibility

No evidence of pain or discomfort was observed, neither edema, hemorrhage, nor dehiscence after the implantation of biomaterials, since there was no evidence that the animals interfered with the surgical wounds. In all animals, surgical wound healing occurred by first intention, and was completed at 15 days postoperatively.

**Fig. 4:**
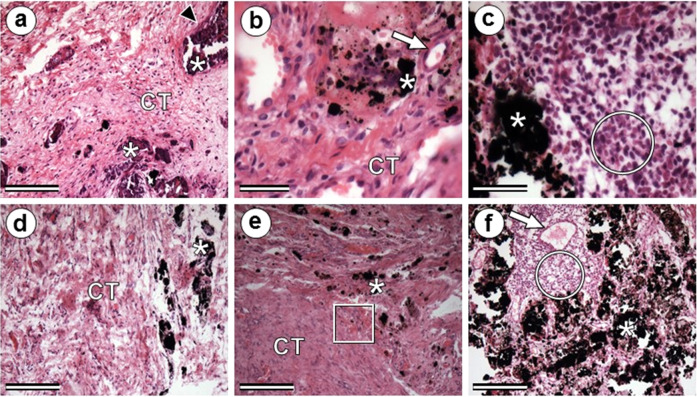
Photomicrographs at 15 (**a** and **b**), 30 (**c**), and 70 (**d**, **e**, and **f**) days after implantation of biomaterials. (**a**) HCoγ_1_; (**b**) HCoγ_2_; (**c**) HCoγ_3_; (**d**) Cγ; (**e**) and (**f**) HC. Asterisk: biomaterial; red arrow: hyperemia; arrowhead: fibrous capsule formation; circle: inflammatory infiltrate; square: bleeding; CT: conjunctive tissue. (**a**), (**c**), (**d**), and (**f**): 100x magnification; (**b**) and (**e**): 400x magnification. HE. Bars: 40 µm

**Fig. 5:**
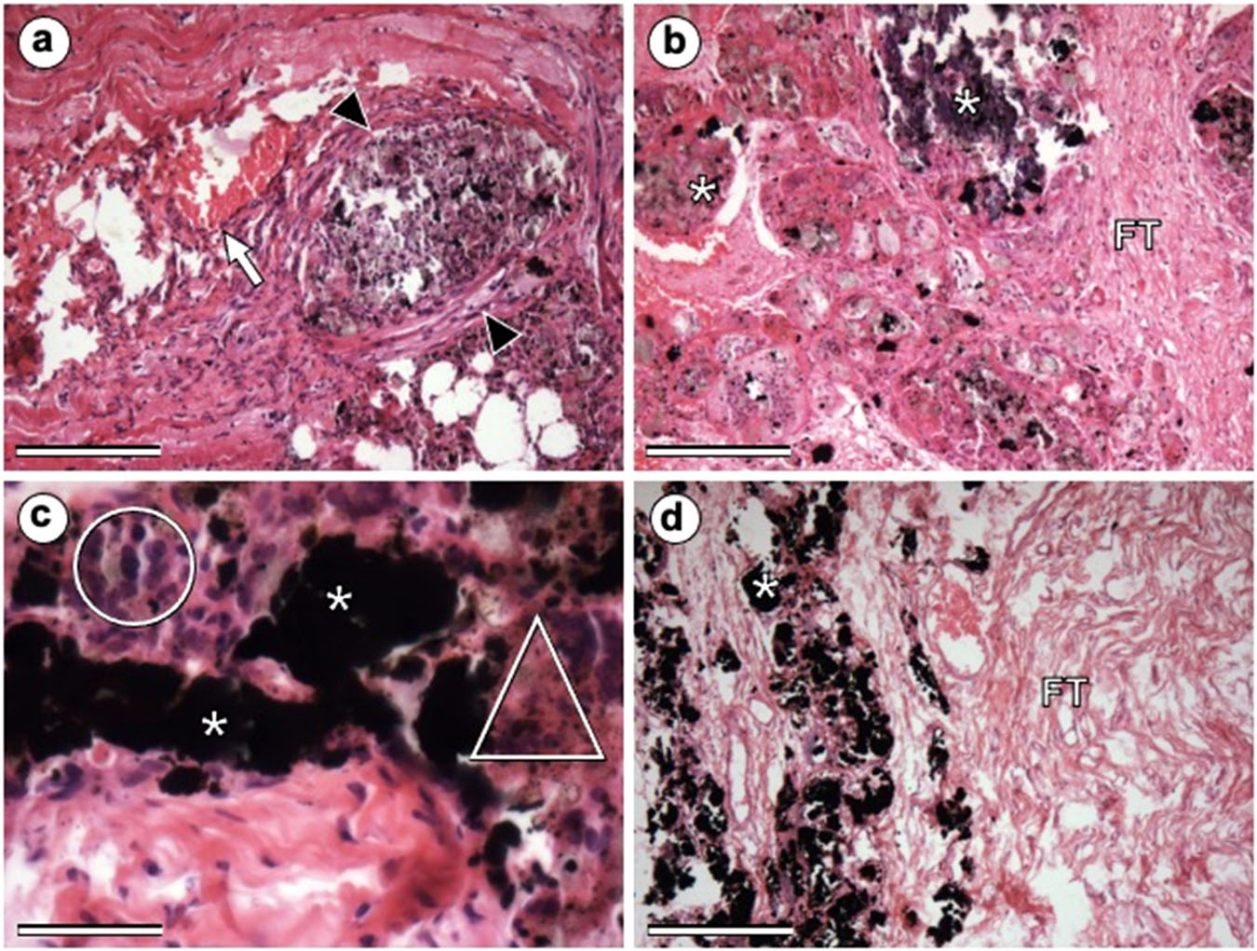
Photomicrographs at 15 (**a**), 30 (**b**), 70 (**c**), and 100 (**d**) days after implantation of the materials. (**a**), (**b**) and (**c**) HC; (**d**) Cγ. Asterisk: biomaterial; arrowhead: fibrous capsule; triangle: hemosiderin deposit; circle: inflammatory infiltrate; FT: organized fibrous tissue (collagen fibers). (**a**), (**b**), and (**d**):100x; (**c**): 400x. HE. Bars: 40 µm

The biomaterials were easily identified during all histological analyses, as an amorphous, granular, acellular, and well-defined material. CoFe_2_O_4_ was visualized in blackish or grayish tones with no affinity for the dye used (HE). HAp exhibited a purplish color, as it has a greater affinity for hematoxylin. In all observations, the biomaterial interacted with the surrounding tissue (Figs. 4 and 5).

At 15 days after implantation of composites, proliferation of inflammatory cells of monocytic lineage was observed around biomaterials. Figure 5b shows this proliferation around the HCoγ_2_ biomaterial. At 30 days, the samples still contained moderate-to-intense amounts of mononuclear inflammatory infiltrate (Fig. 4c), except in the HC treatment, which contained an infiltrate considered discrete to absent (Fig. 5b). Only in this sample, evidence of phagocytosis of the implanted material was observed. At 70 days, the inflammatory infiltrate had disappeared in the HCoγ_1_ sample and was classified as mild in the other samples, except in the HCoγ_3_ sample, which presented moderate infiltration. In the HCoγ_2_, JCoγ, and Cγ samples at 100 days, a discrete inflammatory infiltrate was observed, as described above.

At 15 postoperative days, besides the presence of a moderate-to-intense number of inflammatory cells, it was possible to observe proliferation of connective tissue around the biomaterial (Fig. 4a, b, Fig. 5a). At 30 days, a decrease in fibroblasts could be seen in the newly formed connective tissue. This tissue was more visually organized than at 15 days, that is, the presence of collagen fibers was already noticed. On this date, all samples exhibited a moderate-to-intense degree of fibroplasia. At 70 days, fibroplasia was classified as medium to intense in all samples. At 70 and 100 days, this tissue was more organized and less vascularized. At 100 days, the fibrous tissue showed a more organized appearance, with a predominance of collagen fibers and a reduction in the number of fibroblasts.

At 15 days, it was possible to identify evidence of blood-vessel proliferation in the region implanted in all animals. At 30 days, blood vessels were observed in greater number than in the previous observation, showing neovascularization, classified then as medium to intense, corroborating the studies of Day et al. [32], who describe the stimulation of neovascularization by HAp when implanted in subcutaneous tissue. Only in the JCoγ_1_ sample, hyperemia was classified as mild. In subsequent observations, the degree of hyperemia decreased, until it was no longer evident in HCoγ_1_, HCoγ_3_, and HC treatments at 100 days. This fact is linked to the maturation of the fibrous tissue, which gradually becomes more organized, with the predominance of collagen fibers. The neovascularization observed at implantation sites is expected as part of a natural wound-healing process [33] and is also due to the way in which iron oxides and HAp are degraded in the body [34].

In HCoγ_2_ and HCoγ_3_ treatments, a yellowish-brown pigment (hemosiderin deposition) was observed in the proximities of the biomaterial at 15 and 30 days after implantation, respectively. Hemosiderin was also visualized at 70 days after implantation in HC treatment (Fig. 5c), and in HCoγ_1_ and HCoγ_3_ treatments, hemosiderin accumulations were visualized up to 100 post-surgery days. In the samples obtained at this time, the observed amount of biomaterial was reduced compared with the samples collected at 15, 30, and 70 days.

## Discussion

### Characterization

It can be observed in Fig. 1 and Table 2 that all compounds showed crystalline characteristics, which according to Reis et al. [15], is extremely important, since crystallinity influences in vivo performance by affecting the resorption and mechanical properties of biomaterials.

The presence of peaks with high intensity and high basal width for all reflections is also observed (Fig. 1), demonstrating the crystallinity of the samples and their nanostructure characteristics. However, as the HAp concentration decreases, it is observed that the intensity of the HAp peaks decreases, increasing the intensities of the peaks referring to hybridized MNPs and absence of peaks referring to the drug. The absence of peaks corresponding to the drug agrees with the diffractogram of Fig. 1b of the hybrid CoFe_2_O_4_@SiO_2_:γ (Cγ). The crystal’s size in nanometers, shown in Table 2, is an important feature since the nanostructure characteristics of an implant are related to its surface complexity [35].

The complexity of the surface of each biomaterial, evidenced by the irregularity in the arrangement of hydroxyapatite and cobalt ferrite granules, is an important fact, as it can facilitate cell adhesion on the material. This was observed by Kikuchi et al. [35] using titanium implants coated with calcium phosphate and Carlo et al. [36], who observed the tissue reaction to the implantation of HAp in dog’s subcutaneous tissue.

We can notice in all SEM images, structures composed of heterogeneous and highly agglomerated grains. These characteristics were observed by Sadighian et al. [37] when analyzing magnetite nanoparticles to SEM. Houshiar et al. [38] also observed a complex structure of agglomerated grains and different particle sizes, depending on the way cobalt ferrite was prepared (combustion, precipitation, or coprecipitation) in their work. HAp particles can also come in different shapes, such as needle-shaped in the case of calcined HAp, as well as spherical in appearance [39].

Regarding the porosity of the samples, as reported by Rodrigues et al. [20], Woodard et al. [21], and Carlo et al. [40], the presence of pores is important for the formation of new bone, as they allow the migration and proliferation of osteoblasts and mesenchymal cells. Moreover, the porous structure of hydroxyapatite works as a passive support for vascular neoformation (angiogenesis), making the repair process more dynamic. Chow [41] concluded in his review on calcium phosphate-based biomaterials that the presence of pores can accelerate the process of replacing the material with bone tissue. These conclusions are in line with several studies, including Orr et al. [12] and El-Aziz et al. [11], that highlight the fact that a porous surface considerably improves the union between the implant and bone surfaces while allowing its gradual replacement by bone tissue.

Considering the above, all compounds demonstrated desirable characteristics of a biomaterial for bone implants, as cited by Arabnejad et al. [23] and Reis et al. [15], such as superficial complexity and porosity. These characteristics are known to favor cell adhesion on the material, neovascularization, and therefore the transport of cells and nutrients needed for tissue regeneration [12, 23, 34].

In addition to the tests described in this article, the biomaterials used in this study were previously subjected to characterizations by the manufacturer (LABSMAC Laboratory). The tests performed, Fourier transform infrared spectroscopy (FTIR), surface-area measurement, coercivity, remnant magnetization, and saturation magnetization, are described by Santos et al. [39].

### Agar-diffusion test

Prior to the agar-diffusion test, the study of the conjugation efficacy of the drug to the biomaterials and the kinetics of drug release in vitro of the hybrid materials (HAp:MNPs) was performed by the manufacturer’s laboratory (LABSMAC) and is described in [39]. All the biomaterials analyzed were tested in the presence and absence of the magnetic field, and were effective in releasing the drug in both situations. However, in the presence of the magnetic field, the speed of drug release was lower. Another important factor described by Santos et al. [39] is the amount of HAp present in the biomaterials, which influences the volume of drug retained. This fact is due to the great adsorptive capacity of HAp. In biomaterials where it is present in greater proportion, the volume of drug desorbed after the observation time is lower. In the agar-diffusion test, the presence of HAp did not seem to influence the size of the formed halo (Table 3, Fig. 3).

As reported above, all the compounds showed antimicrobial activity against *S. aureus* evidenced by the formation of the halos around them. This result is in line with that described by Zalneravicius et al. [42] who observed size-dependent antimicrobial activity of CoFe_2_O_4_ MNPs against some microorganisms. Tran et al. [43] also reported antimicrobial activity of iron oxide MNPs against *S. aureus*, suggesting that at 9 nm size, iron oxide MNPs can penetrate the bacterial cell and generate reactive oxygen species. In a 2018 study, Zalneravicius et al. [44] demonstrated the antimicrobial activity of CoFe_2_O_4_ MNPs specifically against *S. aureus*, and described this activity as a function of the Co2+ ion. Annapoorani et al. [45] studied the antibacterial activity of CoFe_2_O_4_ MNPs against fungi (*C. albicans*) and multiresistant bacteria (*E. coli* and *K. pneumoniae*). The authors concluded that CoFe_2_O_4_ diffuses on agar and then exerts inhibitory activity on the growth of fungal and bacterial colonies.

As can be seen in Table 3, biomaterials that contained ciprofloxacin in their composition formed halos larger than 21 mm and, therefore, larger than the halo formed by the paper disk impregnated with the drug and the halo formed by the HC biomaterial. These results suggest that the presence of CoFe_2_O_4_ may have had an antimicrobial effect in addition to that of ciprofloxacin. Ramanavicius et al. [46], who also performed a test similar to the one described here, using paper disks impregnated with MNPs of CoFe_2_O_4_ functionalized with oleic acid (CoFe_2_O_4_@Ole) or L-lysine (CoFe_2_O_4_@Lys), observed the formation of halos indicating inhibitory activity against *C. albicans*. These authors also reported antimicrobial activity of CoFe_2_O_4_@Lys and CoFe_2_O_4_@Ole hybrids against *S.aureus*, *C. parapsilosis*, and *E. coli*. Maksoud et al. [47], working with MNPs of CoFe_2_O_4_, observed antimicrobial potential against several types of bacteria after 24 hours of bacterial incubation in agar dishes. These authors observed that the antimicrobial efficacy of cobalt ferrite is enhanced when these nanoparticles are doped with metal ions, such as Zn. Therefore, the results obtained in the test are according to several publications, such as Ramanavicius et al. [46] and Sanpo et al. [48], which state that MeFe_2_O_4_ ferrites, where Me = Co, Zn, Cu, and Ni, are able to fight human pathogens more effectively than antibiotics.

In the work of Ramanavicius et al. [46] in which a test similar to the one done in this work was described, the measurement of halos was not performed, once their formation already indicates antibacterial activity of the tested materials. Therefore, for the purposes of this study, the simple formation of halos around biomaterials is more important than their diameter. In summary, the test result showed that biomaterials that have ciprofloxacin in their formulation were efficient in releasing the drug, that it is effective in containing the bacterial growth of the *S. aureus* strain used, and also showed the cobalt ferrite antibacterial activity.

### In vivo biocompatibility

The postoperative observations indicated a good acceptance of the compounds, since macroscopically no inflammatory reaction was observed and no rejection of the implanted compounds occurred as well. Carlo et al. [36], Yanagida et al. [49], and Sepúlveda et al. [50] obtained similar results in their studies with Hap associated with bioglass, HAp covering lactic acid, and synthetic HAp for skin filling, respectively.

The implantation of a biomaterial within the living organism induces a series of immune responses collectively referred to as foreign body response (FBR), which attempts to eliminate and/or isolate the implanted material [51, 52]. According to Anderson et al. [53], the initial response to the implantation of a biomaterial is the observation of an acute neutrophilic inflammatory infiltrate, present for up to one week. In addition to neutrophils, other cells involved in the inflammatory reaction induced by implanted biomaterials are lymphocytes, monocytes, and macrophages [54, 55]. The intensity and duration of the inflammatory response is quite varied and is related not only to host-linked factors, but largely to the characteristics of the implanted material, such as surface chemistry, roughness, and porosity [56, 57]. Ye et al. [58] observed after the implantation of HAp in the middle ear of rats, an inflammatory infiltrate with predominance of lymphocytes, followed by neutrophils in smaller amounts. In the present study, because the first observations were made 15 days after the operation, the presence of neutrophils, which characterize the acute phase of the inflammatory process, was not observed.

After this initial period, the so-called chronic inflammatory phase occurs. This phase is characterized by the presence of cells of the monocytic lineage, representing an unspecific inflammatory process, and is confined to the implant site, in case of biocompatibility [59], as in the present study. As explained previously, the inflammatory infiltrate observed around an implant has a variable duration. In this study, the monocytic inflammatory infiltrate was present in some animals until 100 days (Fig. 4e, f, Fig. 5c). However, from 30 days after implantation, it was possible to notice the gradual reduction of inflammatory cells, until their complete disappearance in HCoγ_1_, HCoγ3, and Cγ. In samples in which the inflammatory infiltrate persisted until 100 days (HCoγ2, JCoγ, and HC), it was discrete and restricted to small sections of the slides. This fact was expected due to the biocompatibility of the biomaterials used in this study.

Abudayyak et al. [60], in a study to evaluate the behavior of CoFe_2_O_4_ nanoparticles in contact with mammalian cells in vitro, observed that at certain concentrations (0.1–100 µg/ml), and especially when they are not covered by some biocompatible material,induce toxic responses in general, with oxidative stress, cell death, and exacerbated inflammatory response. In the present study, an inflammatory response was observed, but as previously described, this response was attributed to the surgical procedure to implant the biomaterial. We can infer from this result that the coating of CoFe_2_O_4_ nanoparticles was effective in avoiding the deleterious effects listed above. Moreover, in a normal wound-healing process, an inflammatory reaction, granulation tissue formation, and extracellular matrix deposition occurs initially [33, 61]. Therefore, the inflammatory response observed in this study was considered compatible with what was expected and described in the literature.

Carlo et al. [36] and Sepúlveda et al. [50] observed a large amount of multinucleated giant cells around the HAp particles implanted in the subcutaneous tissue of dogs. In the present work, they were visualized only in HCoγ1 and HC samples, but in small amounts and only at 15 days postoperatively. The foreign body giant cells (FBGC) are the result of the fusion of macrophages present around the biomaterial. They appear when the particles of the biomaterial are too large to be phagocyted by the macrophages. Macrophages and FBGCs induce infiltration and stimulation of immune cells (e.g., lymphocytes) and stromal cells (e.g., fibroblasts), leading to inflammation and fibrosis in the implant [55, 62] with consequent encapsulation. The intensity of this inflammatory reaction and fibrosis is related to many factors linked to the implant, as previously described [56, 57]. Therefore, its presence in small quantities, as in the present study, or even its absence, is an indication of the biocompatibility of the implanted material.

At 15 postoperative days, besides the presence of a moderate-to-intense number of inflammatory cells, it was possible to observe proliferation of connective tissue around the biomaterial (Fig. 5a). This tissue had characteristics of an active tissue, rich in fibroblasts and blood vessels (hyperemia in varying degrees). These findings are characteristic of the normal reaction of the organism when in contact with an implant, prosthesis, or biomaterial [53], and were also observed by Carlo et al. [36], who observed fibroblast proliferation and formation of fibrous tissue around HAp particles implanted in subcutaneous tissue of dogs and by Sepúlveda et al. [50], when implanting HAp in subcutaneous tissue of rabbits. In the HCoγ_1_ sample, this fibrous tissue appeared to be more organized, tending to encapsulate the material. Other authors, such as Borges et al. [19] and Vital et al. [22] observed similar facts and concluded that the formation of fibrous tissue around HAp particles is normal, even when implanted in bone. However, it is important to note that this fibrous tissue formation is more intense when HAp is implanted in soft tissue, with a tendency to encapsulate it [35, 49]. When implanted in bone tissue, HAp usually makes a direct connection with the new bone formed, without the interposition of fibrous tissue [19, 63]. Finetti et al. [64] also observed, after placing a cobalt ferrite implant coated with a polysaccharide hydrogel in the subcutaneous tissue of rats, that after 7 days, there was infiltration of fibrovascular tissue in it. In our study, the proliferation of fibrous tissue around the biomaterial was observed at all times, and it showed to be more disorganized and cellularized, with proliferation of blood vessels, until the first 30 days postoperatively.

At 30 days, all samples exhibited a moderate-to-intense degree of fibroplasia, which according to Kim et al. [65], corresponds to the isolation of a synthetic material when implanted in soft tissues. As described above, fibroplasia around the implanted biomaterials was considered to be average to intense at 70 days post surgery. In addition, a maturation of the fibrous tissue was observed, which showed less vascularization and a greater predominance of collagen fibers at 100 days. This change in the characteristics of the tissue formed around the implanted biomaterials was described by other authors, as Li et al. [66], who measured the thickness of the fibrous capsule formed around HA implants in the subcutaneous tissue of rats, and by Ye et al. [58] in the middle ear.

The tissue organization around a biomaterial implanted in soft tissue, with a progressive tendency to encapsulation, was observed by Li et al. [66], Sepúlveda et al. [50], and Carlo et al. [36]. According to these authors, a complex surface, which is the case of the composites used in this study, may be favorable in bone to promote cell adhesion; however, in soft tissues, the same surface may cause mechanical discomfort to the tissues and, consequently, encapsulation as a defense mechanism. In this study, we observed evident fibroplasia, with a tendency to encapsulate the biomaterial (Fig. 5a). This result is in accordance with that described by Borges et al. [19] and Carlo et al. [36]. According to these authors, HAp resorption in bone is performed by osteoclasts. When it is implanted in soft tissues, it is encapsulated by fibrous tissue and its absorption is slower, being performed by macrophages and foreign body giant cells.

The reduction on the amount of the compounds during the observations is a fact that indicates the degradation of them (Fig. 5d). In addition, the presence of hemosiderin deposits, resulting from iron metabolism by macrophages [67], suggests that CoFe_2_O_4_ is being degraded.

During the experimental times, the degradation and interaction of the composites with the surrounding tissue occurred without causing an evident toxic reaction and without significant inflammatory infiltration. Therefore, they are considered biocompatible. The complexity of the composite surface, observed by SEM, explains some of the events observed in histology. This complexity is extremely important for the use of these materials in bone regeneration, since this characteristic is beneficial to the cell adhesion process. In addition, surface complexity may contribute to biodegradation in a controlled manner, according to Xie et al. [68], especially when it comes to biomaterials implanted in bone tissue, once they must be reabsorbed as new bone is formed.

## Conclusion

It can be concluded that the biomaterials analyzed are suitable to allow tissue growth on them, due to their high surface complexity, evidenced by SEM. XRD showed the high crystallinity of the materials and the presence of the main phase of cobalt ferrite for the CoFe2O4/SiO2 system and the Cγ carrier. The agar-diffusion test showed that they are effective in inhibiting the growth of *S. aureus*, and is therefore indicative of their efficacy as implants in regions infected by this microorganism.

Histological analysis showed that biomaterials are biocompatible. There were no histological signs of rejection by the tissues where they were implanted, or even toxicity, and they induced a controlled inflammatory response. They are biodegradable; however, the degradation period is longer than 100 days.

Thus, based on the obtained results, their subsequent evaluation for bone regeneration in bone infection is indicated.
